# Supplementary material for: Science, interrupted: Funding delays reduce research activity but having more grants helps
Source: PLoS One. 2023 Apr 26;18(4):e0280576. doi: 10.1371/journal.pone.0280576 (PMC10132550; doi:10.1371/journal.pone.0280576)
Supplement: S1 File — (PDF) [file pone.0280576.s001.pdf]

Notes

[1]By policy, the NIH funds projects in annual increments so this places some restrictions on the spending trajectory. The spending trajectory is also shaped by the fact that employee costs, which entail a longer commitment, tend to be the largest costs in a project.

[2]It is also possible that the project is not renewed, either because the renewal application was unsuccessful or the PI chose not to apply for renewal. I focus solely on projects that are eventually renewed by the end of the fiscal year.

[3]The 30-day threshold is chosen to approximate a month. Most funding begins on the first of the month, thus the "arrival" of new grant funding can be thought as occurring on a monthly basis.

[4]I include "R01-equivalent" and "P01" grants in this measure. The Data section elaborates.

[5]The first number is the arithmetic mean of event study estimates after each estimate has been converted from log points to percentage changes.

[6]For longer interruptions, this is more like a U-shape. The Appendix Section contains estimates from specifications that allow the length of an interruption to vary.

[7]A fiscal year is identified by the year in which it ends. E.g. FY 2001 started on 1 October 2000 and ended on 30 September 2001.

[8]Project periods can also be shorter (1-3 years), but this is uncommon.

[9]Ethics statement: This study was approved under The Ohio State University IRBs 2018B0119 and 2013B0413. OSU IRB has determined that this is not research on human subjects.

[10]The final analysis sample within UMETRICS spans 2002 to 2018. An advantage of using data from this period is being able to avoid conflating disruptions from the COVID-19 pandemic with R01 interruptions. Section (Descriptive Statistics) provides summary statistics.

[11]Summary documentation for 2019 UMETRICS data is also available here.

[12]Some transactions in UMETRICS are negative amounts. These can appear for a number of reasons including returns, discounts, reversing a purchase that was wrongly assigned, or money that was unused and refunded. I discuss this more in Appendix Section .

[13]`https://exporter.nih.gov/`

[14]The NIH defines RO1-equivalent grants as follows:"R01-equivalent grants are defined as activity codes DP1, DP2, DP5, R01, R23, R29, R37, R56, RF1, RL1, U01 and R35 from select NIGMS and NHGRI program announcements (PAs) Not all of these activities may be in use by NIH every year." (Link)

[15]A P01 can roughly be thought of as a combination of R01 projects.

[16]Author names cannot be used directly to identify individuals because multiple versions of the same author's name may appear in the literature (e.g. "Adam Smith" and "A. Smith") and multiple authors may have the same name.

[17]An example is described in the appendices of [16]. [17] provides an accessible description of the method and comparisons with other estimators.

[18]The resulting panel spans 1985 to 2011, where 1985 is the earliest year ExPORTER covers and 2011 is the latest year available to construct three-year forward citations (since WoS only indexes citations up to 2013).

[19]For "large" outcomes i.e. spending amounts, I convert estimates to percentage changes using the standard $exp(\hat{\beta}) - 1$ for log transformations. More details in Appendix Section .

[20]Appendix Section , Figure 13, includes results from matching on NIH IC and university in addition to project length. The results remain similar.

[21]The full set of UMETRICS occupation codes is: Faculty, Research, Graduate Student, Postgraduate, Research Facilitation, Undergraduate, Technical Support, Clinical, Instructional, Other, and Other Staff.

[22]Appendix Section contains more summary statistics about lab size.

[23]This is calculated as the average percentage change from months -3 to 12, weighted by each month's share of total spending over the entire period for the control group.

[24]`https://www.niaid.nih.gov/grants-contracts/caveats-consider-preaward-spending`

[25]One reason may be that it is harder to justify how spending after a grant expires benefits a research project, as suggested in this document from the University of Washington.

[26]Event study estimates for different interruption lengths are available in the Appendix Section .

[27]Note that the "Number of R01s" variable is defined differently for each outcome variable, thus the subsamples used in the analysis are not identical.

[28]One suggestive piece of evidence is that for interrupted faculty, the absolute probability (i.e. not relative to uninterrupted employees) of being paid by a grant increases almost monotonically with time from expiry, whereas interrupted employees in all other occupations hit a plateau even after an initial increase. This may reflect that for faculty (many of whom would be a PI on the R01) the convergence is due to a "recovery" while for other occupations it is due to a "catching up" of uninterrupted employees to their interrupted counterparts.

[29]Appendix section contains additional results exploring heterogeneous effects by NIH IC and PI career age. These also do not indicate an effect of interruptions on publications.
