# Supplementary material for: Science, interrupted: Funding delays reduce research activity but having more grants helps
Source: PLoS One. 2023 Apr 26;18(4):e0280576. doi: 10.1371/journal.pone.0280576 (PMC10132550; doi:10.1371/journal.pone.0280576)
Supplement: Attachment — Submitted filename: interruptions_response-to-reviewers.pdf [file pone.0280576.s002.pdf]

Response to Reviewers

Reviewer 1 comments and responses

1. Temporary nature, and aggregate effect: The effects seem temporary; but when the expenditures rebound, the do not rise high enough to compensate. Can we quantify, the overall change in expenditures (i.e., the stock, not flow) over the two years, starting 3 months before the interruption? This would help us get a take-home number on the long-run reduction in spending by the lab because of the renewal delay.

Thank you for the suggestion to calculate the change in the stock of expenditures, which I agree is also of interest in addition to the change in the flow of expenditures. Using the sample of uninterrupted labs, I take the period starting from three months before interruption to 12 months post-interruption. I calculate the total percentage change in spending over this period as the average monthly percentage change (i.e. the exponentiated point estimates) weighted by each month's share of total spending. This comes up to 52%.

2. Event-studies literature: There is a long new literature on event-studies specifications. The author has cited the literature (Goodman-Bacon 2018; Callaway and Sant'Anna 2018; Abraham and Sun 2018; Borusyak and Jaravel 2017), but it would be great to see the methods applied. The code is readily available on their websites, so implementation isn't too difficult. The author also says "The Online Appendix discusses these in detail", but I had trouble finding it there. It would be good to see the event studies using the methods cited (Callaway and Sant'Anna 2018; Abraham and Sun 2018; Borusyak and Jaravel 2017), or in fact using the method of the stacked-regression in the appendix of the Cenzig, Dube, Lindner, Zipperer (2019) QJE paper.

Thank you for bringing up this point. I agree with the reviewer that the connection to the new literature on difference-in-differences could have been clearer and I have now

updated the paper to reflect that. In the section explaining the specification for estimating the effect of interruptions on spending, I have added the paragraph below:

> An important literature on misspecification of the two-way fixed effects model for the staggered difference-in-differences design has emerged in recent years (Goodman-Bacon 2021; Callaway and Sant'Anna 2021; Sun and Abraham 2021; Borusyak, Jaravel, and Spiess 2021). Compared to the typical setting considered in this literature, one advantage of the setting in this paper is being able to define a "post-treatment" period for control units, i.e., the expiry date of the R01 grant. This means that we can define time relative to treatment for both treatment and control units. Thus, the above specification estimates an event study for a difference-in-differences with only one treatment period *in relative time*, avoiding the key issue raised in the literature.

As an alternative, I have also re-estimated the main results using the Callaway-Sant'anna estimator (instead of OLS) and included the results in the Appendix, under the section "Callaway-Sant'anna estimator". I include them here as well for convenience (Figure 1).

In the event study for publications, I was already using the stacking procedure (e.g. Cengiz et al. (2019)). However, I have now made that connection clearer in the exposition.

3. Three months pre-trends and selection into delay: The fall in expenditures begins 3 months before the interruption. Can we have a short discussion about why 3 months before?

Thanks for highlighting that this was not explained clearly. In the *Results* section of the original manuscript, I state that:

> . . . Since we observe a divergence in spending even before the official expiry date, this indicates that PIs have some ability to shift spending outside of the official budget periods. This can happen in two ways.
>
> The first way is that PIs can engage in "pre-award spending", where they make a request to incur costs 90 days before the official start date of the grant. Thus, if PIs of uninterrupted grants know early enough that they will be funded, some of the divergence in spending could be due to them making use of pre-award

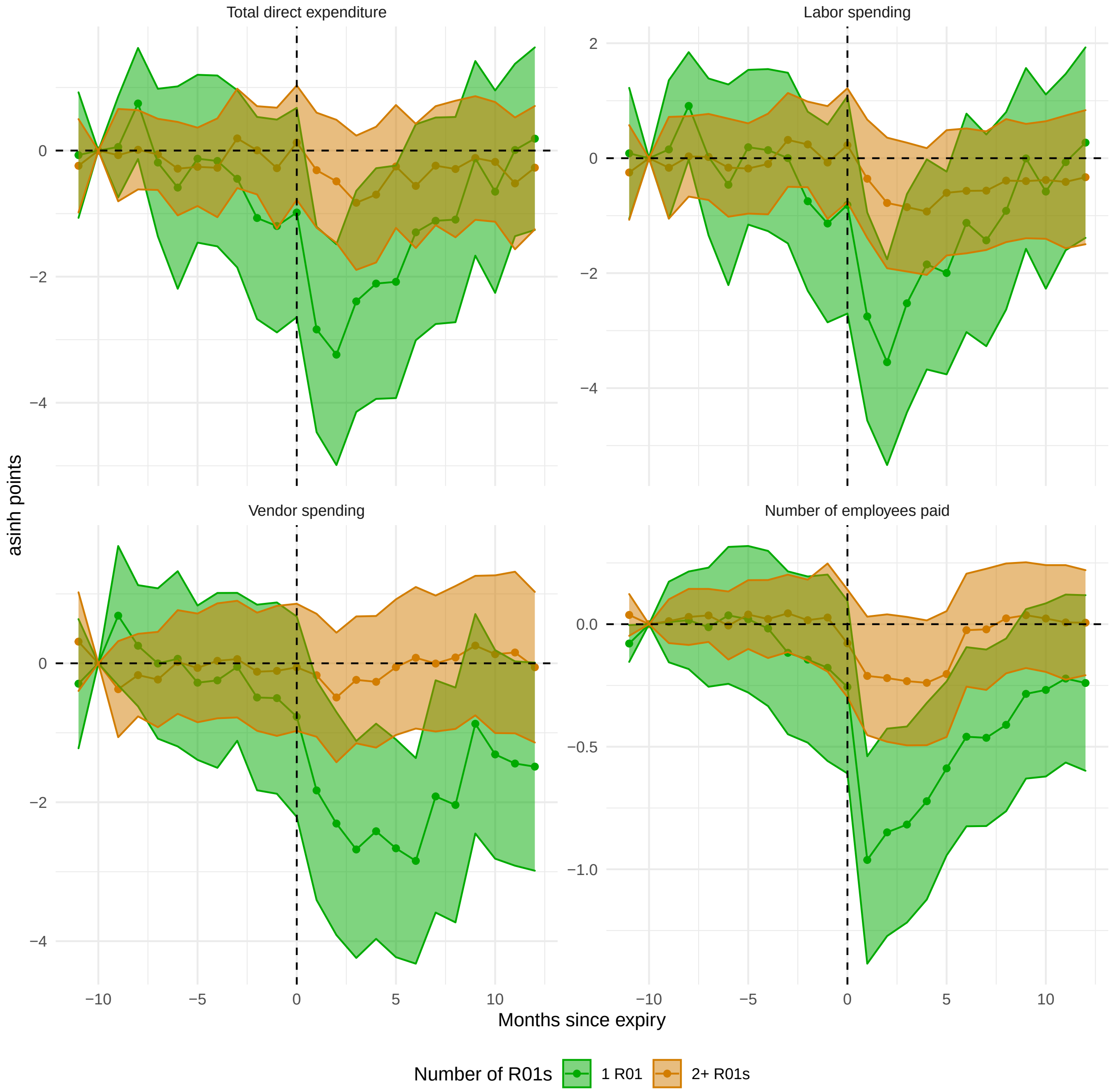

Figure 1: This figure shows event study estimates (with 95% confidence intervals) of the difference in spending between PIs of interrupted and uninterrupted R01s. Estimation is done using the Callaway-Sant'anna (2020) doubly robust estimator, including the length of the expiring R01 project period as a covariate. Each panel shows the estimates for a different outcome variable: total expenditure by PI, total vendor expenditure by PI, total labor expenditure by PI, and total number of employees paid by PI. Month 0 is the month that the focal R01 expires. Month -10 is the excluded category for the regression. For comparison with the main results, the point estimates and confidence intervals have been adjusted so that the point estimate for Month -11 is 0. Regressions are run separately on subsamples of PIs that have one R01 grant (green) or multiple R01s (brown), including R01-equivalents and P01 grants. Standard errors are clustered at the expiring R01 level.

> spending.
>
> The second way is that PIs of interrupted grants may be be able to delay spending beyond the official end date of the grant. For example, they might want to hold off on hiring a postdoctoral researcher until there is more certainty that they have the funding to support them. While these actions are not mutually exclusive, a university administrator said that in their experience, pre-award spending was used "quite often", whereas saving funds for later was not common.

However, I was not clear about the implication and have now added an additional statement to be clear about what the takeaway from this discussion is:

> Based on these remarks and the timing of the divergence in spending, the first explanation (pre-award spending by uninterrupted grants) seems to be the more likely reason for what we observe.

4. Minor: The first regression equation has δ_{LR} fixed effects, but the text following it discusses it as δ_{iR} (employee level instead of PI level). I believe it's just a minor typo.

Thank you for catching this typo. The notation in the text was wrong and has been corrected.

5. Minor: Online Appendix: Many times the text refers the reader to the Appendix, but it is hard to find where in the appendix we should look at. I suggest saying "refer to Online Appendix Section A.3" or "Figure A.4 in the Online Appendix".

I appreciate this helpful suggestion to make it easier on the reader. The main text now includes references and links to specific sections of the Appendix.

Reviewer 2 comments and responses

1. The analysis is well done and compelling. The missing component, however, is what the practical impact is of the large decline in both working employees and vendor spending. Take the employees, for example. If graduate students are released from labs for some number of months, are they truly

not working? If so, how does that impact the career success of those graduate students? Are they more likely to not graduate if they had that disruption in their work? Or, do graduate students continue to work in the labs but without compensation—creating the dip you find in your analysis, but without an impact on the training of the graduate students themselves? Figure 4 begins to address this, but teasing out which of these stories is correct is important since in some the impact is fairly limited while in others the impact is substantial and would be a call for additional policy response.

Thank you for this point. I agree that understanding the impact on graduate students (and other employees) is crucial. Since labs are the main unit of production in the biomedical sciences, this paper's focus is on understanding how labs/PIs respond to funding interruptions and whether interruptions might affect lab production of research.

In a follow-up paper with coauthors (in progress), we are looking into the question of how funding interruptions affect careers. Tracking individual labor market outcomes requires substantial additional work (e.g. adding data sources like US tax data, the Decennial Census, and publications linked to *individuals* rather than PIs/labs). Thus, we determined that studying the labor market implications was beyond the scope of this paper and required more careful consideration in a follow-up paper.

2. The empirics are generally clear and appropriate. The question that I would like the paper to address is why you used the control group that you chose for the analysis. One might imagine running similar analysis using the same labs but during an earlier time period as a control. You could also imagine restricting the control group to be within the same university or field. I would suggest that the motivation for the choice of control group and the reason for dismissing other potentially valid control group specifications be made explicit in the paper.

Thank you for this suggestion. The original manuscript was missing some detail on the choice of control group and specification. At the referee's suggestion, I have now added the following paragraph to be explain the choice of control group:

> The goal is to estimate how outcomes for labs/PIs change when they experience an R01 interruption. In doing so, it is important to take into account the decrease

> in spending observed at the end of a grant. . . Thus, a natural comparison group for labs with interrupted R01s is labs with R01s are at similar stages in the cycle, that is, labs with R01s that were also expiring but were successfully renewed.

". . . using the same labs but during an earlier time period as a control": I take this remark to mean restricting the sample to eventually-treated units and running either an interrupted time series or using comparisons of treated units to not-yet-treated units. I think this would be a less ideal strategy for this particular context. One reason is the relatively small number of eventually-treated units. For example, the initial sample size of interrupted PIs with one R01 is 72, and quickly becomes too small as we match on more variables and/or include heterogeneity analyses. The other reason (as I now discuss in the quoted paragraph above) is the importance of comparing the expenditure patterns of grants at similar stages. My current approach of centering time on the R01 expiry date and then "stacking" the units makes it transparent that the comparisons are being made between R01s at similar stages and straightforwardly avoids the usual pitfalls of the staggered difference-in-differences.

"You could also imagine restricting the control group to be within the same university or field": I interpret this as a sugggestion to match on more variables. Figure 2 below shows a set of results where I match on project period length (as requested before), university, and NIH Institute & Center (aka NIH IC, which I believe is what the referee was referring to as "field"). The sample size is much smaller after matching but the overall takeaway of the results does not change. I have included these results in the Appendix as well.

3. It is not clear what the aggregate impact of funding delays is on science. If scientists use less of their funds when the funding is delayed, they will presumably be able to spend more later to make up for that dip and expend the entirety of their grant. In the event study plots, however, it is not clear where the labs are spending more than their steady-state level. Could you please explain why we don't see higher than average spending after the dip is done? Could you also try to estimate what is the total impact over the course of the grant if labs spend less in the period when funding is delayed and more in later periods?

Thank you for raising these interesting points.

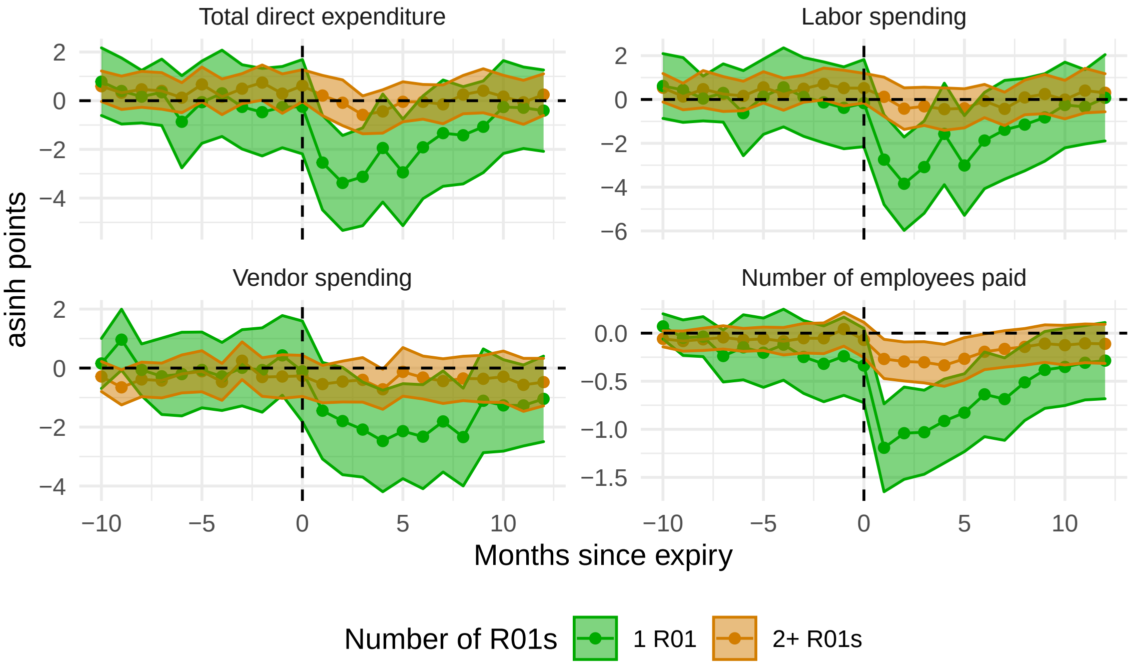

Figure 2: This figure shows event-study estimates (with 95% confidence intervals) of the difference in spending between PIs of interrupted and uninterrupted R01s. Treated and control groups are matched on length of the expiring R01 project period, NIH IC, and university, and the regression is weighted using the matching weights. Each panel shows the estimates for a different outcome variable: total expenditure by PI (A), total vendor expenditure by PI (B), total labor expenditure by PI (C), and total number of employees paid by PI (D). Month 0 is the month that the focal R01 expires. Month -11 is the excluded category for the regression. Regressions are run separately on subsamples of PIs that have one R01 grant (green) or multiple R01s (brown), including R01-equivalents and P01 grants. Standard errors are clustered at the expiring R01 level.

Could you please explain why we don't see higher than average spending after the dip is done?

It is not a given that interrupted PIs will spend above their steady-state level, or at least not necessarily in a way that would be obviously detectable. PIs are not "saving" during the dip in spending — they simply do not have access to NIH funds. Thus, when the funds become available, the PI still has to ensure that they have enough funding to last till their next grant. For example, suppose a PI hires one graduate student in the "steady state". If the PI could not hire a graduate student for several months due to an R01 interruption, it is unlikely that they would have the budget to hire two graduate students to make up for the lost time. Since labor costs make up the majority of a lab's expenditure, this places some restrictions on how much of an "overshooting" we might observe. In addition, the nature of their research may be such that it is not easy to make up for lost time simply by spending more money (e.g. time may be the binding constraint, rather than person-hours).

Could you also try to estimate what is the total impact over the course of the grant if labs spend less in the period when funding is delayed and more in later periods?

As requested, I extend the event study till the end of the renewed grant (Figure 3). The panel ends at month 72 i.e. the final month of a 6-year grant. The panel is not balanced because (1) grants last for different lengths of times and (2) sample attrition due to the transaction data for different schools spanning different dates.

It is difficult to conclusively say whether there is more spending in the later periods. We can at least rule out that spending "overshoots" in the same way that it falls just after the R01 expires. Total spending towards the end of the graph is steadily higher although by that point the sample size is also much smaller (for the two reasons mentioned above).

4. The most notable heterogeneity is the difference between labs with one versus multiple R01 grants. Could you provide additional information about why labs are able to compensate for delays using other R01s but are not able to compensate with other non-R01/departmental funds? Relatedly, could please provide a figure that shows that PIs with multiple R01s increase their spending on their other R01 to compensate for the decreased spending on the delayed R01 (if true). If you do not

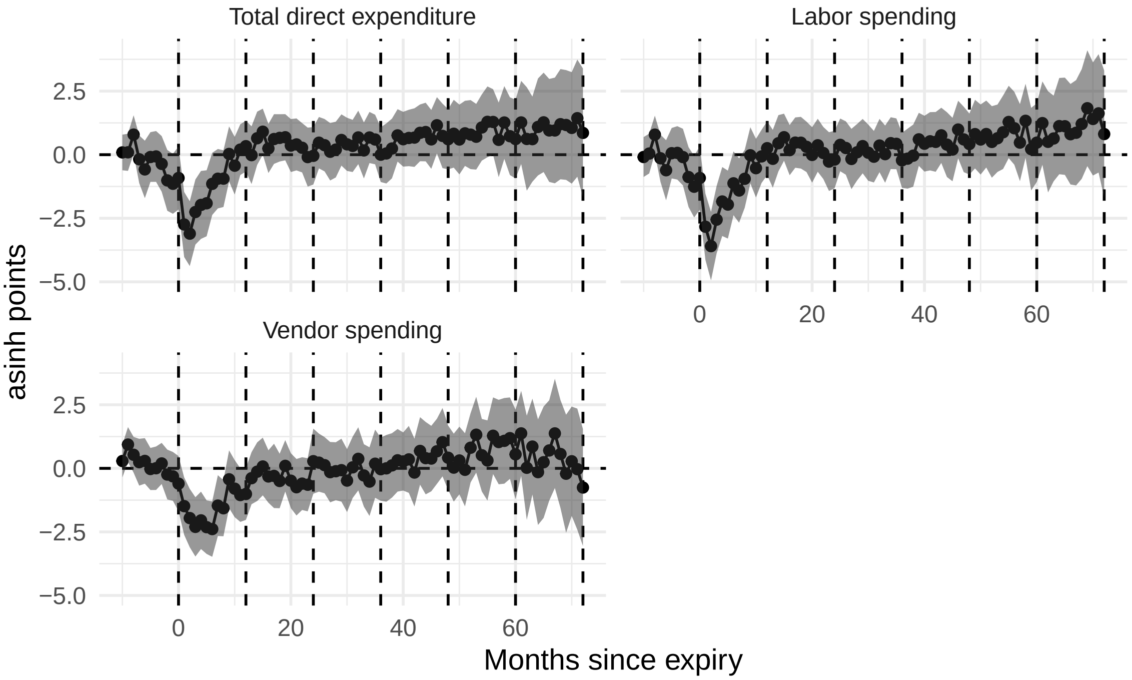

Figure 3: This figure shows event-study estimates (with 95% confidence intervals) of the difference in spending between PIs of interrupted and uninterrupted R01s. The panel extends till the end of the renewed R01 or the end of . Each panel shows the estimates for a different outcome variable: total expenditure by PI, total vendor expenditure by PI, total labor expenditure by PI. Month 0 is the month that the focal R01 expires. Month -11 is the excluded category for the regression. Regressions are run separately on subsamples of PIs that have one R01 grant (green) or multiple R01s (brown), including R01-equivalents and P01 grants. Standard errors are clustered by expiring R01 project period.

find a commensurate increase in expenditures on the unaffected second R01 grant then why are PIs with multiple grants able to maintain their level of spending?

Could you provide additional information about why labs are able to compensate for delays using other R01s but are not able to compensate with other non-R01/departmental funds?

Thank you for the question and opportunity to clarify. The UMETRICS data captures spending from *sponsored projects* (i.e. grants). Thus, spending from funding sources such as departmental funding or bridge funding from the university are not observed in the data I use, but PIs could certainly be drawing on them. When I calculate a PI's spending, it is based on the *total spending from NIH grants* associated with the PI. Thus, any spending from grants other than the delayed R01 is spending from NIH grants that could be R01s (except the delayed R01) but could also be other kinds of non-R01 NIH grants.

...provide a figure that shows that PIs with multiple R01s increase their spending on their other R01...

As requested, Figure 4 below shows the median amount spent each month in the year before and after R01 expiry for the sample of PIs with (1) an interrupted R01 and (2) multiple R01-equivalents. I use the median here because of the skewed distribution of spending. As the reviewer suggested, we see a spike in spending from the PI's remaining grants around and after the focal R01 is interrupted.

5. Additional exploration of heterogeneity by fields, size of lab, PI age, and other attributes could be very interesting to readers. For example, Appendix Section 1.3 clearly shows heterogeneity in the rates of interruptions across fields. Similarly, heterogeneity in the impact on employees by employee attributes could be of interest to policymakers.

Thank you for this suggestion. I have included additional heterogeneity analyses for the analysis looking at lab publications, which allows for a larger sample. I explore heterogeneity by NIH Institute and Center (also known as ICs, which the reviewer refers to as "fields") and by career age (time since first publication). In the Appendix Section D.4.3, I have now included the next two figures. The first figure (Figure 5) shows estimates

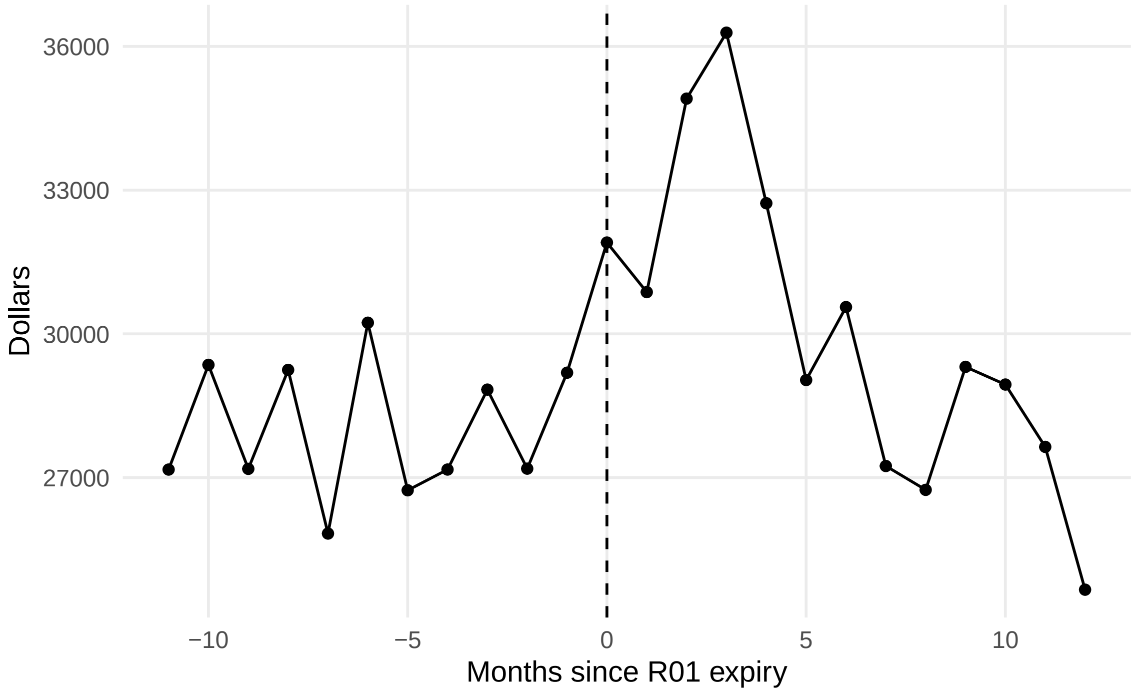

Figure 4: Median spending from grants *other than* the expiring R01 for PIs/labs with multiple R01s and that had an interrupted R01.

of the effect of interruptions on publication counts by IC. These are obtained by running separate regressions on each IC subsample.[1] Similarly, the second figure (Figure 6) shows estimates obtained by running separate regressions by whether the PI was above or below the median sample career age (defined as years since first publication). In all cases, the estimates are statistically insignificant.

Heterogeneity by employee attributes (e.g. gender, occupation) is certainly interesting as well. As discussed in the earlier response, we are working on a new paper focused on the effects of interruptions on employees that will explore these heterogeneous impacts on a richer set of outcome variables (for employees) than were available for this paper.

6. Appendix Section 1.2 says that the time series of interruptions provide evidence that the NIH is responsive to delays in the federal budgeting process. Could the author please expand on how the figure demonstrates this?

Thank you for pointing out the need to clarify this. My intention was simply to highlight

[1]Since ICs vary in size, I limit the set of ICs to those with at least 100 PIs in the treatment group (since that is smaller than the control group).

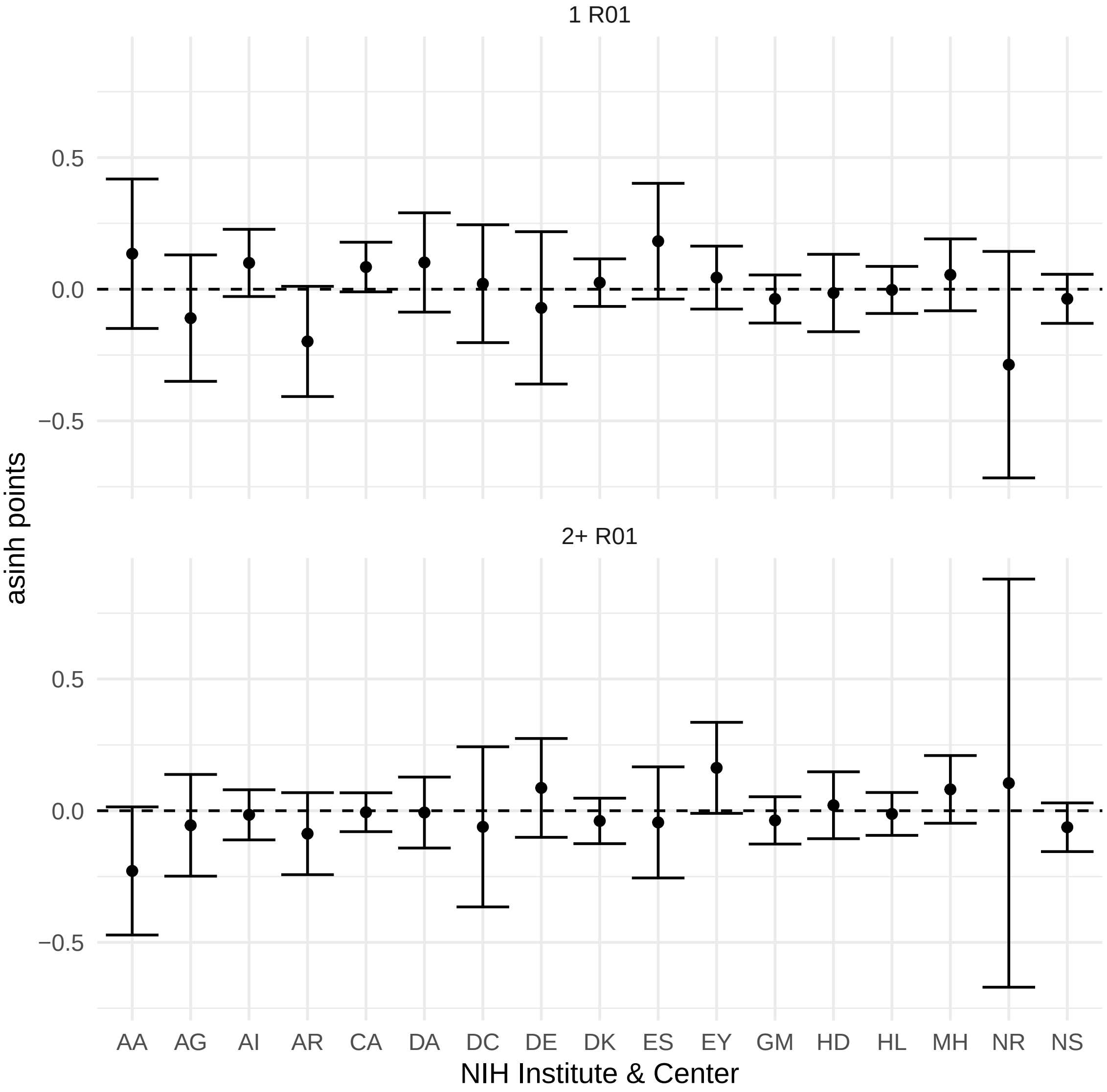

Figure 5: This figure shows "static" difference-in-difference estimates and 95% confidence intervals of the difference in publication output if a PI had an interrupted R01, estimated separately on NIH IC subsamples. The regression includes treatment-cohort-by-year and PI-R01-renewal fixed effects. Dependent variables are raw publication counts, arcsinh-transformed. Standard errors clustered at the PI-R01-renewal level.

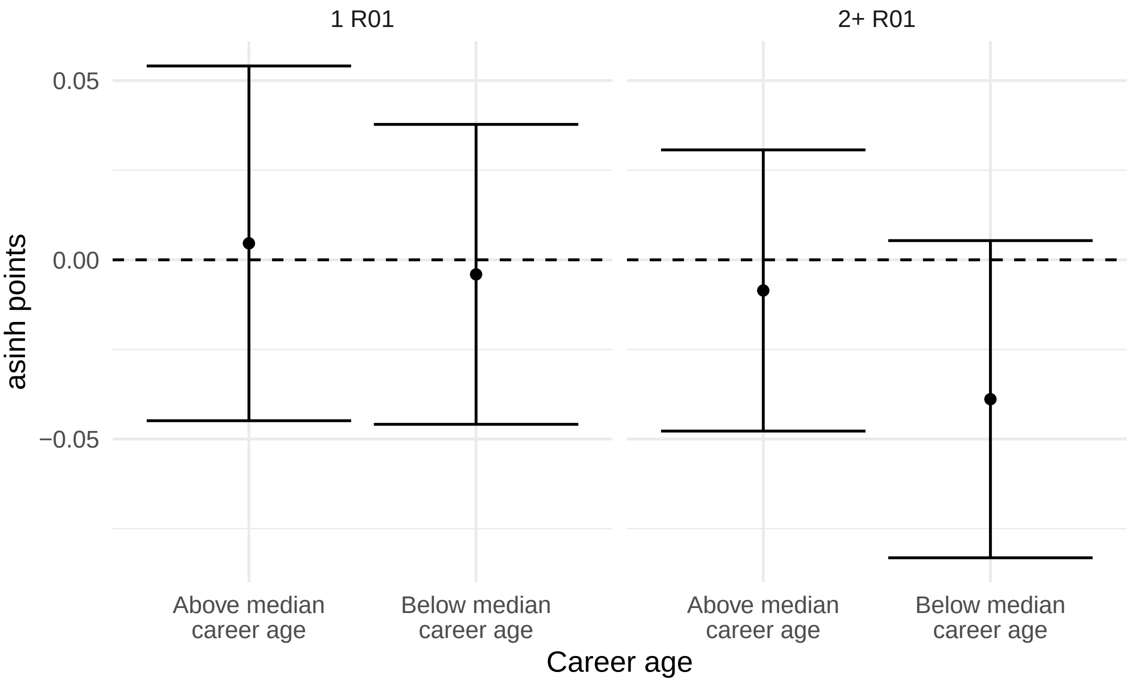

Figure 6: This figure shows "static" difference-in-difference estimates and 95% confidence intervals of the difference in publication output if a PI had an interrupted R01, estimated separately on subsamples divided by whether the PI was above or below the median career age in the sample. The regression includes treatment-cohort-by-year and PI-R01-renewal fixed effects. Dependent variables are raw publication counts, arcsinh-transformed. Standard errors clustered at the PI-R01-renewal level.

Fiscal Year 1996 (when there was a three-week shutdown) as an example of how disruptions in the grant-making process might affect the rate of interruptions. I have re-written that section to be clearer about about this point. The text now reads as:

> There is an increase in the rate of interruptions over time, though with substantial fluctuations around the overall trend. I also highlight the spike in interruptions in Fiscal Year 1996 — when there were two federal government shutdowns (including one that lasted three weeks) that were reported to be highly disruptive to the grant making process (Mervis and Marshall 1996) — as suggestive that the NIH does respond to delays in the federal budgeting process.

7. The author sometimes refers to labs. Can the author please clarify if a single PI is equivalent to a "lab" or if a lab can have multiple PIs?

Thank you for pointing out this potential area of confusion. I use the terms "lab" and "PI" interchangeably. This is now made clear in the second paragraph of the introduction.

> I use UMETRICS to study how Principal Investigators (abbreviated as PIs; for the remainder of the paper, I use the terms "lab" and "PI" interchangeably) funded by the National Institutes of Health (NIH) respond to funding delays or "interruptions".

8. Why is the preferred specification in the main text not matched on project period length and the matched version in Appendix 3.1.1 instead of the other way around? I find the matched project length version more compelling, however, if there is a reason to prefer the unmatched version, I would ask the author to note that in the empirical framework section.

Thank you for this suggestion. I've now made the matched project length specification the main one discussed in the text.

9. What is the difference between the employees with the title "Research" and the title "Research Facilitation"?

"Research" staff are directly involved in conducting research but are not faculty members

(e.g. technicians, staff scientists, statisticians)

"Research Facilitation" refers to employees who are not specifically employed for scientific research but who perform job tasks that support the research enterprise, such as coordinating clinical trials or managing a large facility (e.g. administrative staff, lab coordinator (but not lab manager), grants manager, project manager).

I have also added to the Appendix the complete list of occupations in UMETRICS along with detailed descriptions.

References

Borusyak, Kirill, Xavier Jaravel, and Jann Spiess. 2021. "Revisiting Event Study Designs: Robust and Efficient Estimation." *arXiv Preprint arXiv:2108.12419*.

Callaway, Brantly, and Pedro HC Sant'Anna. 2021. "Difference-in-Differences with Multiple Time Periods." *Journal of Econometrics* 225 (2): 200–230.

Cengiz, Doruk, Arindrajit Dube, Attila Lindner, and Ben Zipperer. 2019. "The Effect of Minimum Wages on Low-Wage Jobs." *The Quarterly Journal of Economics* 134 (3): 1405–54.

Goodman-Bacon, Andrew. 2021. "Difference-in-Differences with Variation in Treatment Timing." *Journal of Econometrics* 225 (2): 254–77.

Mervis, Jeffrey, and Eliot Marshall. 1996. "Science Budget: When Federal Science Stopped." *Science* 271 (5246): 136–36.

Sun, Liyang, and Sarah Abraham. 2021. "Estimating Dynamic Treatment Effects in Event Studies with Heterogeneous Treatment Effects." *Journal of Econometrics* 225 (2): 175–99.
