# Supplementary material for: Science, interrupted: Funding delays reduce research activity but having more grants helps
Source: PLoS One. 2023 Apr 26;18(4):e0280576. doi: 10.1371/journal.pone.0280576 (PMC10132550; doi:10.1371/journal.pone.0280576)
Supplement: Attachment — Submitted filename: interruptions_response_round2.pdf [file pone.0280576.s003.pdf]

Response to Reviewers

Thank you for the opportunity to address Reviewer 2's comments and improve the paper. I've written brief responses below, leaving out Comments 6 and 7, which were about typos that I have now fixed.

Reviewer 2 comments and responses

1. The main text data section could be a clearer on the sample timeframe. Specifically, am I correct that the data on employment goes from 2001 to 2018, but the research output is measured for 2001 to 2013? The main data section mentions the raw databases that are linked together to form the analytical dataset, but it would be helpful if the authors also described the main analytical dataset, including what time period is actually covered for the outcome variable and covariates in the main text.

I've now added information about the timeframes of the analytical sample to the Data section so that it is available where the reader would naturally look, with links to later sections for more details. To directly address the reviewer's question: the UMETRICS source data is from 2001 to 2018, while the sample derived from it is from 2002 to 2018. The data for analyzing research output is from 1985 to 2011.

I've also added Figure 1 to the Appendix showing the years in which expiries occurred in the UMETRICS and ExPORTER analysis samples so that readers have a sense of the treatment timing.

2. Some of the most interesting results from this paper could be the heterogeneity across research and subfield of research. I would recommend bringing Appendix D.4.3 into the main text and expanding on what can be learned from it. The heterogenous effects on young researchers are particularly interesting; is this evidence of a Matthew Effect? I am less clear on what the conclusion is regarding

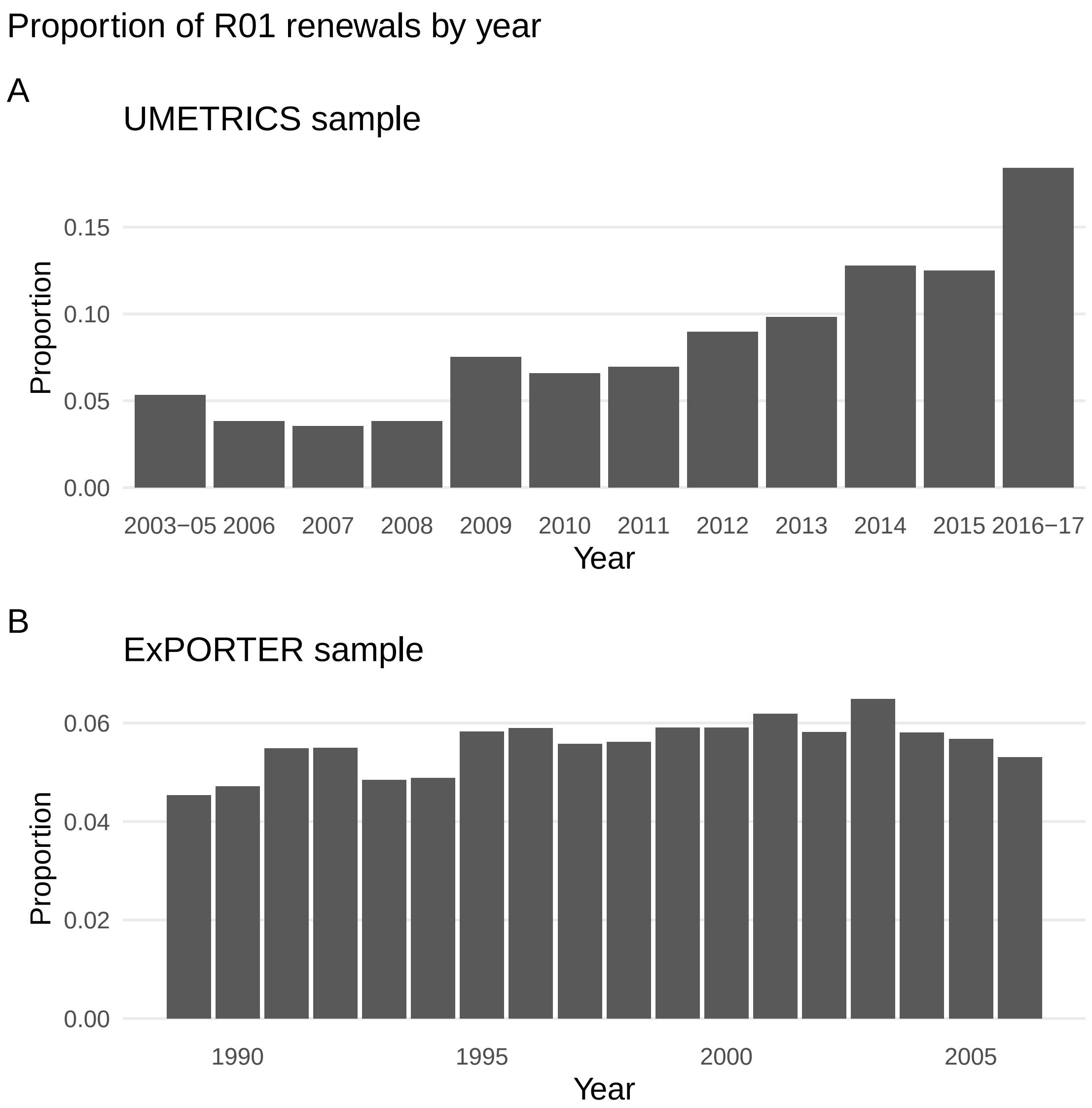

Figure 1: This figure shows the timing of R01 expiry for each PI-R01 combination in the analysis samples. Figure A shows the proportion of PI-R01s from the UMETRICS sample where expiry occurred in a given year, while Figure B shows the same for the ExPORTER sample.

the heterogeneity across NIH centers, but would appreciate if the authors could expand on it.

Given that the estimates from the research output analyses are statistically insignificant from zero in all cases, my takeaway from the totality of the heterogeneity results is that there is insufficient evidence to conclude that there is an effect of interruptions on research output. As a result, I would prefer to leave those results as is.

3. I am still a little confused about the aggregate spending impact. If the total spending change is 52% decline, where did the remaining money go? I understand that a researcher might forgo hiring a graduate student, and thus the researcher will have lower labor costs. But does that mean that the researchers are simply not spending down their full grants now? If data is not available to address this, I would look qualitative evidence or comments from PIs regarding how they handle the excess funds at the end of their grant.

The total spending change is calculated over a 16-month period (starting from three months before R01 expiry, when the decrease in spending begins, to 12 months after R01 expiry), not the entire cycle of the grant. I have now made sure that any use of the "52%" statistic is clear that it only applies to the 16-month window.

4. Figure 2(b) – This graph is very useful for understanding if there is a sharp distinction between those with less than a 30 day and those with more than 30 day interruption. Could you please bin the days of interruption, such that we can see with more granularity the distribution under ~60 days?

I believe the reviewer is asking for a more granular histogram of the gap between expiry and renewal for R01s that were not interrupted. I have now added Figure 2 to the Appendix.

5. I appreciate the inclusion of Figure 6 showing the rate of interruptions over time. My one concern is that the timeline ends at 2014, but you are using data through 2018. Even if you filtered to PIs with a year's worth of monthly data following the disruption, I would expect this graph to go through 2017. Could you please either update that figure or explain why it is cut at 2014?

I have updated Figures 5 and 6 to go up to and including 2020.

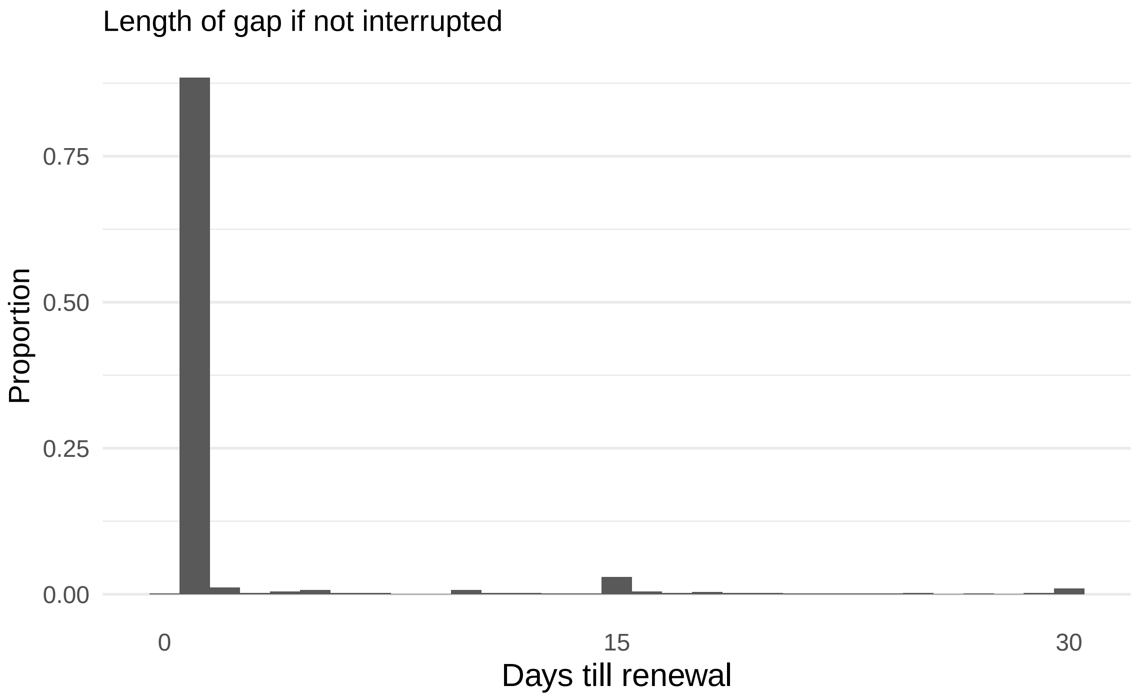

Figure 2: This figure shows the distribution of the time between expiry and renewal for R01 project periods that were not interrupted, i.e., renewed within 30 days. Each unit of observation is an R01 project period. The figure is a histogram with 1-day bins.

8. Given the lack of impact on research outcomes and yet the massive impact on employee counts (particularly for single R01), is there some way to tell if the labs employees are simply switching to uncompensated work? For example, can you see if the names of the researchers on the publications from the labs change when the PIs reduce the employment during the interruption? If you could show that there was uncompensated work done, this would be a contribution to policy debates about grad student and postdoc compensation.

This is an important point that we hope to shed more light on in our follow-up paper on the effects of interruptions on labor market outcomes.

9. The authors use the UMETRICS data through 2018. I imagine that the reason for not expanding into more recent years is because the authors do not want to conflate funding disruptions with the COVID-19 disruptions. I would encourage the authors to mention the motivation for the sample time frame chosen in the main text.

I used the 2019 release of UMETRICS, the latest available data at the time of the analysis. However, the reviewer's point about COVID-19 is also a good one and I have noted it in

the Data section as well.
